# The Role of Primary Cilia in Modulating the Luteinization Process of Ovarian Granulosa Cells in Mice

**DOI:** 10.3390/ijms26052138

**Published:** 2025-02-27

**Authors:** Xiaochuan Long, Xiayu Min, Xinyao Xiao, Yao Wu, Zengming Yang, Xin Wen

**Affiliations:** 1 Clinical Veterinary Laboratory, College of Animal Science, Guizhou University, Huaxi District, Guiyang 550025, China; 15086027944@163.com (X.L.);; 2 Key Laboratory of Animal Genetic, Breeding and Reproduction in the Plateau Mountainous Region, Ministry of Education, Guizhou University, Huaxi District, Guiyang 550025, China; 3 Basic Veterinary Laboratory, College of Animal Science, Guizhou University, Huaxi District, Guiyang 550025, China

**Keywords:** luteinization, primary cilia, progesterone, insulin

## Abstract

The corpus luteum is the principal progesterone-secreting gland, while primary cilia function as pivotal organelles in intercellular signal transduction. Together, they play an essential role in the establishment and maintenance of pregnancy. However, the mechanisms underlying the role of primary cilia in granulosa cell luteinization in mouse ovaries remain poorly understood. This study discovered the regularity of primary cilia in mouse ovaries and revealed the role of primary cilia in regulating progesterone synthesis in luteinized granulosa cells. In vivo test results showed that the expression of primary cilia was obvious in the corpus luteum. The secretion of P4 in mice was significantly increased at 6, 12, 24, 48, and 72 h. The secretion of P4 and the expressions of luteinization markers (STAR, 3β-HSD) and primary ciliate proteins (IFT88, Arl13B) were significantly up-regulated at different time points (6, 12, 24 h and 24, 48, 72 h), and the INS group was significantly higher than the LH group and the control. In vitro test results showed that the follicular granulosa cells were luteinized under INS, the length and number of primary cilia increased, and the secretion of progesterone increased. The expression levels of STAR and 3β-HSD of the primary cilia marker Arl13B and luteinization markers were increased, while the expression levels of CYP19A1 were decreased. Ciliobrevin A (CBA) and Y-27632 2HCl were used to regulate the expression of primary cilia. The results showed that after CBA treatment, the expression level of cilia protein Arl13B decreased, and the secretion level of P4 and the expression levels of STAR and 3β-HSD decreased, indicating that the level of luteinization decreased. Conversely, after inducing ciliogenesis with Y-27632 2HCl, the results were the opposite of those observed with CBA treatment. In conclusion, our study demonstrates that primary cilia regulate the expression of steroidogenic enzymes, thereby promoting progesterone secretion by granulosa cells in mice and ensuring proper luteinization.

## 1. Introduction

The ovary is a vital organ in the mammalian reproductive system, playing a crucial role in sustaining reproductive health and ensuring normal fertility [1]. During the physiological cycle, ovarian follicles undergo a series of developmental and maturation processes, with luteinization representing a critical stage. The corpus luteum is formed from somatic cells, including theca cells and granulosa cells, within the follicle following ovulation, playing a pivotal role in the maintenance of pregnancy [2]. As the primary gland responsible for the secretion of progesterone (P4) and estrogen (E2) post-ovulation, it is essential for ovarian endocrine function [3,4,5,6]. The theca cells differentiate into small luteal cells (SLCs), while granulosa cells undergo luteinization to give rise to large luteal cells (LLCs) [2,7]. The formation and functional maintenance of the corpus luteum are contingent upon the precise proliferation and differentiation of granulosa and theca cells [2]. However, the molecular mechanisms underlying the regulation of luteinization in ovarian granulosa cells remain incompletely understood.

Luteinization represents a profound transformation in both the morphology and function of the ovarian follicle. Prior to ovulation, under the regulatory control of the hypothalamic–pituitary–gonadal (HPG) axis, the pituitary gland secretes the luteinizing hormone (LH), triggering an LH surge [8,9]. The LH surge induces both ovulation and the formation of the corpus luteum. Under the influence of the LH surge, granulosa cells undergo differentiation into large luteal cells (LLCs), with concomitant molecular changes, such as the upregulation of cytochrome P450 family 11 subfamily A1 (CYP11A1) and 3β-hydroxysteroid dehydrogenase (3β-HSD) [10,11]. These enzymes are critical for the conversion of cholesterol into pregnenolone and subsequently progesterone (P4) [12,13,14]. Moreover, the expression of the steroidogenic acute regulatory protein (STAR) is upregulated, whereas the expression of cytochrome P450 family 19 subfamily A1 (CYP19A1), a key enzyme in estrogen synthesis, is downregulated [15]. Steroid hormones, including P4 and E2, are synthesized when STAR facilitates the transport of cholesterol into the mitochondria, where it is converted to pregnenolone and further processed by cytochrome P450 family enzymes (CYP11, 17, 19) and 3β-HSD [16]. Following the LH surge, these rapid alterations in the expression of STAR and CYP19A1 contribute significantly to progesterone production, a process critical for ovulation and the subsequent luteinization process [17]. Hydroxysteroid 17-beta dehydrogenase 2 (HSD17B2) is an epithelial cell-secreted enzyme that catalyzes the conversion of estradiol to estrone, playing a pivotal role in maintaining hormonal homeostasis. Previous studies have suggested that the increase in STAR expression and the concomitant decrease in CYP19A1 expression are markers of luteinization [17,18,19,20]. Given that the appearance of STAR is spatially and temporally aligned with progesterone synthesis, the upregulation of 3β-HSD, HSD17B2, and STAR expression, along with the downregulation of CYP19A1, can serve as reliable markers for luteinization [12,17,21].

Primary cilia act as essential cellular signal hubs, facilitating the reception of both extracellular chemical and mechanical signals, and are pivotal in regulating homeostasis and various physiological processes in mammals [22,23]. Granulosa cell luteinization is intricately regulated by hormones and growth factors circulating in the bloodstream [24,25]. Therefore, it remains essential to investigate whether primary cilia are involved in signal transduction during follicular granulosa cell luteinization in response to hormonal stimulation. The intraflagellar transport protein (IFT88) and ADP-ribosylation factor-like protein 13B (Arl13B) are indispensable for ciliary assembly and are critical for mediating Hedgehog (Hh) signaling [26,27]. Arl13B, a protein that is exclusively localized to the ciliary membrane, functions as a ciliary-specific marker, facilitating the precise localization of cilia. In recent years, primary cilia, recognized as fine cellular surface structures, have attracted considerable attention from researchers. Primary cilia have been demonstrated to play pivotal roles in a variety of cell types, such as neurons, renal tubular cells, and chondrocytes [28,29,30]. Previous research has confirmed the presence of primary cilia on granulosa cells in mouse ovaries [31]. Furthermore, it has been shown that both progesterone and estrogen collaboratively regulate the formation of primary cilia [32,33]. Estrogen has been shown to inhibit primary ciliary length, while progesterone promotes elongation; the ciliary length in the combined estrogen and progesterone treatment group was intermediate, though the exact function remains undetermined. During the luteal phase, granulosa cell differentiation, along with the synthesis and secretion of estrogen and progesterone, plays a critical role in the physiological processes involved in luteinization [34]. However, the presence and potential role of primary cilia in the luteinization of ovarian granulosa cells in mammals remain to be explored.

This study utilizes the mouse model to investigate the structural changes of primary cilia during granulosa cell luteinization and to assess their impact on the luteinization of mouse granulosa cells. The findings aim to provide more comprehensive experimental data to further elucidate the molecular mechanisms underlying corpus luteum formation, while also offering new insights into the prevention and treatment of miscarriages due to luteal insufficiency.

## 2. Results

### 2.1. Expression Changes of Primary Cilia During the In Vivo Induced Luteinization of Mouse Granulosa Cells

#### 2.1.1. Primary Cilia Are Localized to Luteal Cells in Mice

**Figure 1:**
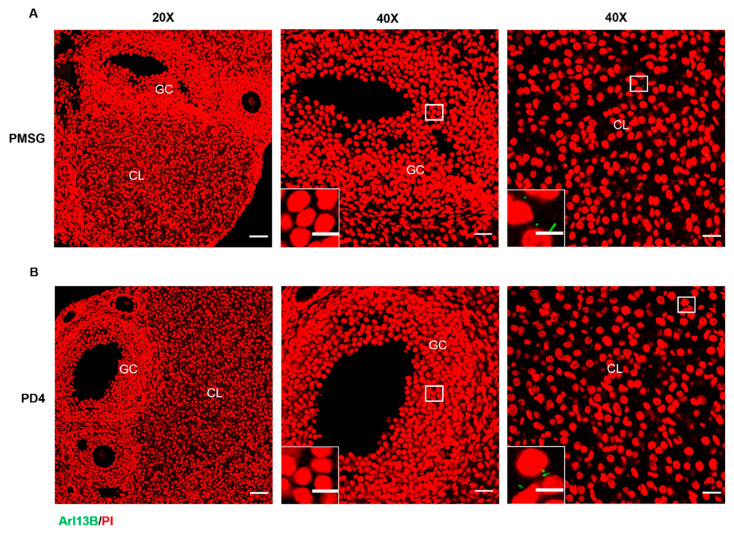
The expression of primary cilia in mouse ovaries. (**A**) Immunostaining of the ciliary marker Arl13B in the ovaries of PMSG-injected mice. GC: granulosa cells, CL: corpus luteum. Nuclei were counterstained with PI. Scale bars: 50 μm (main panel) and 10 μm (insets). (**B**) Immunostaining of the ciliary marker Arl13B in the ovaries of pseudo-pregnant mice on day 4 (PD4). GC: granulosa cells, CL: corpus luteum. Nuclei were counterstained with PI. Scale bars: 50 μm (main panel) and 10 μm (insets).

Ovaries were collected from PMSG-induced follicular development mice and from pseudo-pregnant mice on day 4 (PD4), and the expression and localization of the ciliary marker Arl13B (a membrane marker of primary cilia) in the ovaries were analyzed. Immunofluorescence analysis revealed that Arl13B in both the PMSG-treated group (Figure 1A) and the PD4 group (Figure 1B) was predominantly localized to the ovaries and corpora lutea, with comparatively lower expression in the follicles. These findings suggest that Arl13B likely plays a critical role at specific stages of ovarian development, particularly during luteinization.

#### 2.1.2. Th Upregulation of Primary Cilia Expression During the In Vivo Induced Luteinization of Mouse Granulosa Cells

**Figure 2:**
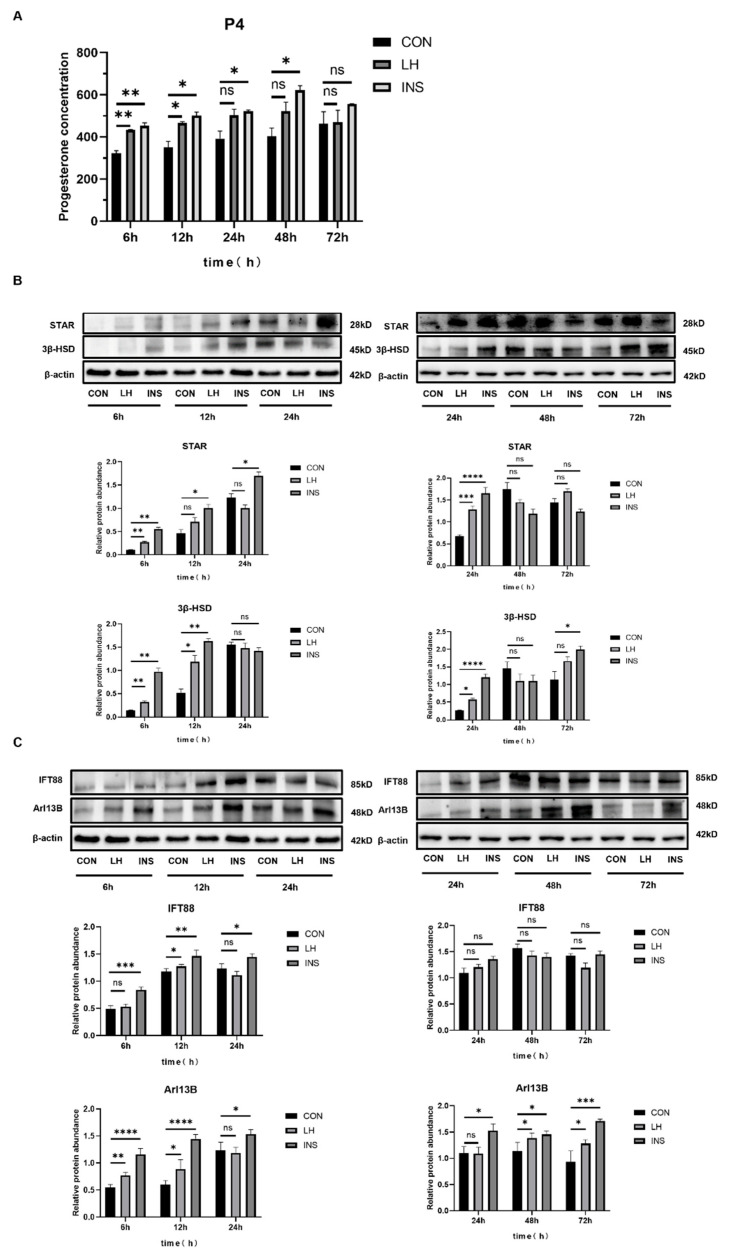
ELISA and Western blot results of in vivo induced luteinization in mouse granulosa cells. (**A**) Serum progesterone levels at 6, 12, 24, 48, and 72 h after injection of physiological saline, luteinizing hormone (LH), and insulin (INS) in mice. (**B**) Western blot analysis of STAR and 3β-HSD expression at 6, 12, 24, 48, and 72 h after injection of physiological saline, LH, and INS in mice. (**C**) Western blot analysis of IFT88 and Arl13B expression at 6, 12, 24, 48, and 72 h after injection of physiological saline, LH, and INS in mice. * *p* < 0.05, ** *p* < 0.01, *** *p* < 0.001, **** *p* < 0.0001, ^ns^ *p* > 0.05.

The experiment was conducted with three groups: the control group (CON), which received physiological saline; the luteinizing hormone (LH) group; and the insulin (INS) group, with time points at 6, 12, 24, 48, and 72 h. Serum and ovarian tissues were collected to assess the levels of P4 secretion and the expression of related proteins. ELISA analysis revealed that P4 secretion levels were significantly higher in both the LH and INS groups compared to the CON group (Figure 2A). Western blot analysis demonstrated that at 6, 12, 24, 48, and 72 h after LH and INS injections, the expression of luteinization markers STAR and 3β-HSD was progressively upregulated in the INS group compared to the CON group. At each time point, the INS group exhibited significantly higher expression levels than the CON group, while the LH group showed intermediate levels (Figure 2B). Western blot analysis of primary cilia markers IFT88 and Arl13B revealed that, compared to the CON group, IFT88 expression was significantly elevated in the LH and INS groups at 6, 12, and 24 h, with no significant changes at 24, 48, and 72 h. Arl13B expression was significantly elevated at all time points (6, 12, 24, 48, and 72 h), with higher levels in the INS group than in the LH group (Figure 2C).

Taken together with the results from Section 2.1.1 and Section 2.1.2, Arl13B, a ciliary marker, is highly expressed in large luteal cells and minimally expressed in granulosa cells. During the luteinization process induced by INS or LH, the expression of primary cilia markers IFT88 and Arl13B, as well as P4 secretion levels, were positively correlated. It is hypothesized that the formation of primary cilia may influence the luteinization of granulosa cells, a hypothesis that was further confirmed in subsequent experiments.

### 2.2. Patterns of Primary Cilia Formation During the Luteinization of Mouse Granulosa Cells

#### 2.2.1. The Length and Number of Primary Cilia Increased in the Early Stage of Luteinization of Mouse Follicular Granulosa Cells

Based on previously reported methods for establishing luteinization models [35,36], we employed insulin (INS) to construct an in vitro luteinization model using mouse granulosa cells. After treating granulosa cells with different concentrations of insulin (INS), immunofluorescence analysis was conducted to assess cilia length and number (Figure S1A). The results showed that under stimulation with 2 μg/mL of INS, the length of primary cilia in luteinizing granulosa cells was significantly greater than in the 1, 4, and 8 μg/mL INS-treated groups (Figure S1B). Furthermore, the number of cilia was also significantly higher in the 2 μg/mL INS-treated group compared to the 1, 4, and 8 μg/mL INS-treated groups (Figure S1C). Analysis of progesterone (P4) secretion revealed that under stimulation with 2 μg/mL of INS, P4 secretion was significantly higher compared to the 1, 4, and 8 μg/mL INS-treated groups (Figure S1D).

**Figure 3:**
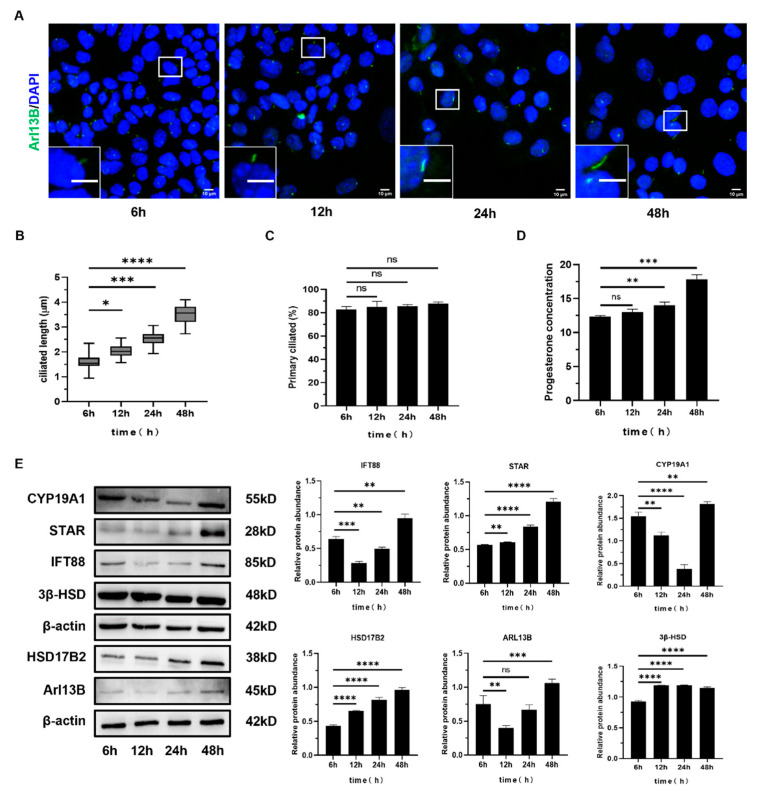
The expression of primary cilia during the early stages of luteinization in mouse granulosa cells. (**A**) Immunofluorescence staining of granulosa cells treated with insulin (INS) for 6, 12, 24, and 48 h. Cell nuclei were stained with DAPI. Scale bars: 10 μm (insets and main panel). (**B**) The length of primary cilia in granulosa cells treated with INS for 6, 12, 24, and 48 h. (**C**) The percentage of primary cilia per granulosa cell following INS treatment for 6, 12, 24, and 48 h. (**D**) Progesterone secretion levels in granulosa cells treated with INS for 6, 12, 24, and 48 h. (**E**) Immunoblots of STAR, 3β-HSD, HSD17B2, IFT88, Arl13B, and CYP19A1 in granulosa cells treated with INS for 6, 12, 24, and 48 h. All data are expressed as the mean ± SD of three independent experiments, with representative images shown. Statistical comparisons between two groups were performed using a two-tailed *t*-test. * *p* < 0.05, ** *p* < 0.01, *** *p* < 0.001, **** *p* < 0.0001, ^ns^ *p* > 0.05.

Based on these results, an insulin concentration of 2 μg/mL was selected for subsequent experiments. After 6, 12, 24, and 48 h of INS treatment, ELISA and immunofluorescence analysis (Figure 3A) showed a gradual increase in primary cilia length (Figure 3B), with no significant change in the cilia number (Figure 3C). Additionally, P4 secretion exhibited a gradual increase over time (Figure 3D). Furthermore, Western blot analysis revealed that, after 6, 12, 24, and 48 h of 2 μg/mL INS treatment, the expression of luteinization markers STAR, 3β-HSD, and HSD17B2, along with primary cilia proteins IFT88 and ARL13B, was significantly increased, while the expression of CYP19A1 was significantly decreased (Figure 3E). These findings suggest that insulin stimulation promotes the luteinization of granulosa cells, and that primary cilia may play a crucial role in this process.

#### 2.2.2. The Length and Number of Primary Cilia Decreased During the Late Luteinization of Mouse Follicular Granulosa Cells

**Figure 4:**
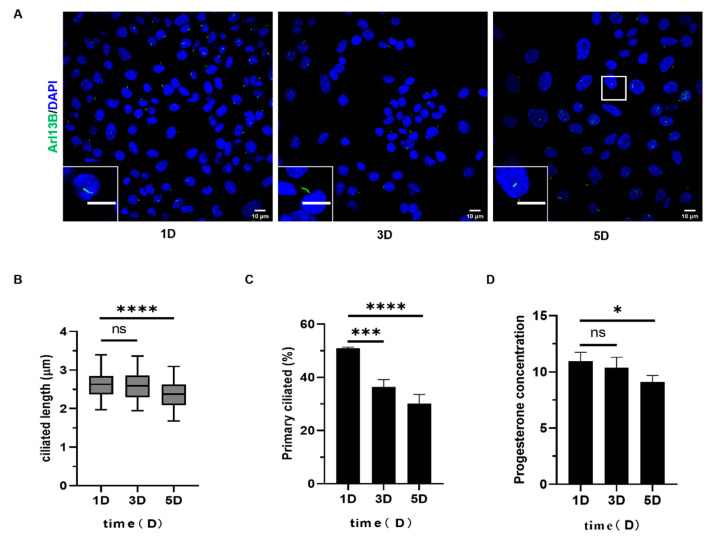
The expression of primary cilia during the later stages of granulosa cell luteinization in mice. (**A**) Immunofluorescence staining of granulosa cells after 1, 3, and 5 days of INS treatment. Nuclei were stained with DAPI. Scale bar: 10 μm (insets and main panel). (**B**) The primary cilia length in granulosa cells after 1, 3, and 5 days of INS treatment. (**C**) The percentage of primary cilia per granulosa cell after 1, 3, and 5 days of INS treatment. (**D**) Progesterone secretion levels in granulosa cells after 1, 3, and 5 days of INS treatment. All data are presented as the mean ± SD from three independent experiments, with representative images shown. Statistical analysis was performed using a two-tailed *t*-test for comparisons between the two groups. * *p* < 0.05, *** *p* < 0.001, **** *p* < 0.0001, ^ns^ *p* > 0.05.

Prolonged INS treatment of granulosa cells revealed no significant changes in primary cilia length or number in the 1-day and 3-day treatment groups. However, in the 5-day treatment group, the length of primary cilia was significantly reduced (Figure 4A,B), the cilia number decreased significantly (Figure 4C), and progesterone secretion (P4) also exhibited a declining trend (Figure 4D). Combined results from Section 2.2.1 and Section 2.2.2 show that during the early stages of luteinization, the length and number of primary cilia increased in parallel with the progesterone-driven luteinization of granulosa cells. In contrast, during the later stages of luteinization, cilia gradually shortened, indicating that primary cilia play a role in regulating granulosa cell luteinization. The length and number of primary cilia dynamically change throughout granulosa cell luteinization and represent one of the regulatory factors involved in this process.

### 2.3. Effect of Dynamic Changes of Primary Cilia on Luteinization of Follicular Granulosa Cells in Mice

#### 2.3.1. The Expression of Luteinizing Related Molecules and the Secretion of Progesterone in Mouse Follicular Granulosa Cells Decreased After Inhibiting the Occurrence of Primary Cilia

**Figure 5:**
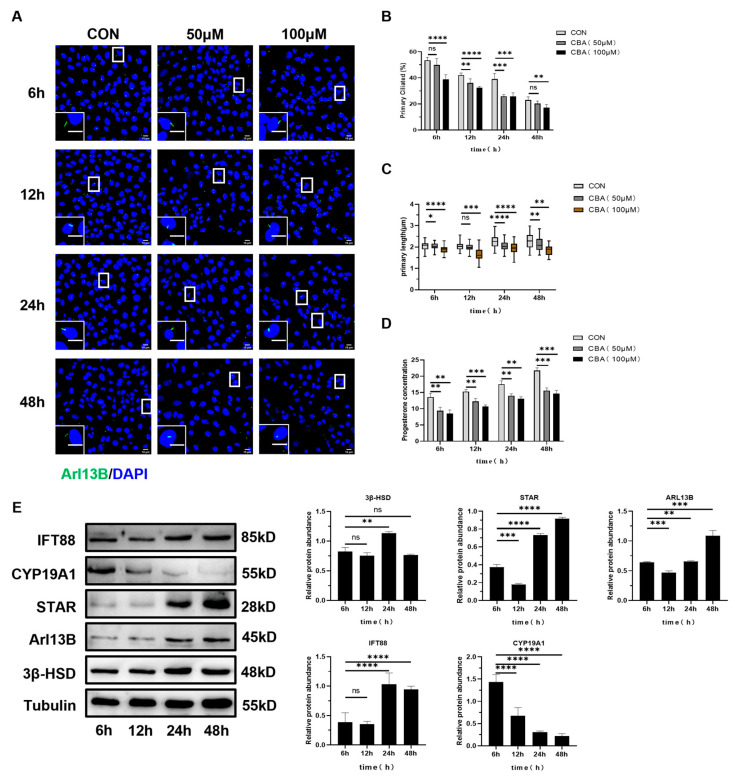
The effects of different concentrations of Ciliobrevin A (CBA) on the number and length of primary cilia and progesterone synthesis in luteinized granulosa cells. (**A**) Immunofluorescence staining of granulosa cells treated with 50 μM or 100 μM of CBA for 6, 12, 24, and 48 h. Cell nuclei were stained with DAPI. Scale bar: 10 μm (insets and main panel). (**B**) The percentage of primary cilia in granulosa cells treated with 50 μM or 100 μM of CBA for 6, 12, 24, and 48 h. (**C**) The length of primary cilia in granulosa cells treated with 50 μM or 100 μM of CBA for 6, 12, 24, and 48 h. (**D**) Progesterone secretion levels in granulosa cells treated with 50 μM or 100 μM of CBA for 6, 12, 24, and 48 h. (**E**) Western blot analysis of STAR, 3β-HSD, IFT88, Arl13B, and CYP19A1 in granulosa cells treated with 50 μM or 100 μM of CBA for 6, 12, 24, and 48 h. All data are presented as the mean ± SD of three independent experiments, with representative images shown. Comparisons between the two groups were made using a two-tailed *t*-test. * *p* < 0.05, ** *p* < 0.01, *** *p* < 0.001, **** *p* < 0.0001 ^ns^ *p* > 0.05.

To investigate the role of primary cilia in regulating granulosa cell luteinization, we employed Ciliobrevin A (CBA), a ciliogenesis inhibitor, to suppress primary cilia formation. Immunofluorescence (Figure 5A) and ELISA results demonstrated that treatment of granulosa cells with 50 μM and 100 μM of CBA significantly reduced the percentage of ciliated cells (Figure 5B) and the primary cilia length (Figure 5C), with a concomitant decrease in progesterone secretion levels (Figure 5D). Furthermore, the inhibitory effect of 100 μM of CBA was more pronounced than that of 50 μM of CBA. Immunofluorescence (Figure 4A) and ELISA results demonstrated that the treatment of granulosa cells with 50 μM and 100 μM of CBA significantly reduced the percentage of ciliated cells (Figure 5B) and primary cilia length (Figure 5C), with a concomitant decrease in progesterone secretion levels (Figure 5D). Furthermore, the inhibitory effect of 100 μM of CBA was more pronounced than that of 50 μM of CBA. Western blot analysis revealed that after 6, 12, 24, and 48 h of treatment with 100 μM of CBA, the expression of primary cilia markers Arl13B and IFT88, as well as luteinization markers STAR, 3β-HSD, and HSD17B2, were elevated, while CYP19A1 expression was reduced (Figure 5E).

**Figure 6:**
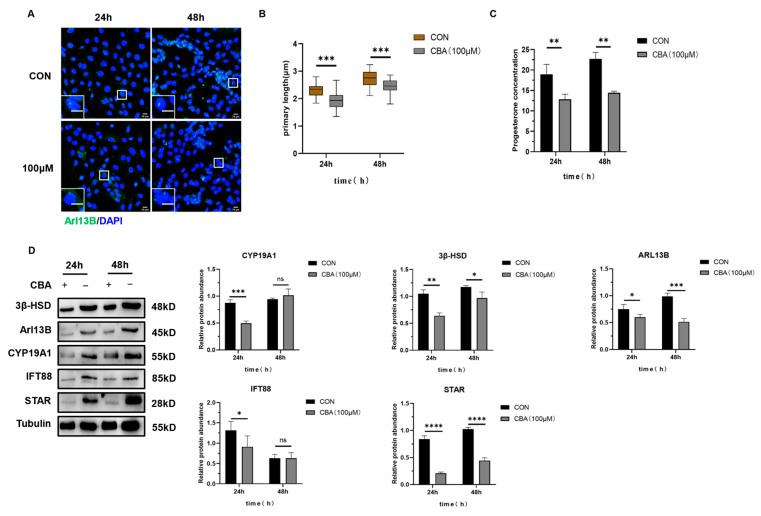
The impact of inhibiting primary cilia formation on the luteinization of mouse granulosa cells. (**A**) Immunofluorescence staining of granulosa cells treated with 100 μM of Ciliobrevin A (CBA) for 24 and 48 h. The cell nuclei were stained with DAPI. Scale bar: 10 μm (insets and main panel). (**B**) Progesterone secretion levels in granulosa cells treated with 100 μM of Ciliobrevin A (CBA) for 24 and 48 h. (**C**) Length of primary cilia in granulosa cells treated with 100 μM of Ciliobrevin A (CBA) for 24 and 48 h. (**D**) Immunoblotting analysis of STAR, 3β-HSD, IFT88, Arl13B, and CYP19A1 expression in granulosa cells treated with 100 μM of Ciliobrevin A (CBA) for 24 and 48 h. All data are presented as mean ± SD from three independent experiments, and representative images are shown. Statistical comparisons between groups were made using a two-tailed *t*-test. * *p* < 0.05, ** *p* < 0.01, *** *p* < 0.001, **** *p* < 0.0001, ^ns^ *p* > 0.05.

Granulosa cells were treated with 100 μM of Ciliobrevin A (CBA) for 24 and 48 h. The results showed that compared to the control group, the secretion of progesterone (P4) was reduced (Figure 6B), and the length of primary cilia shortened (Figure 6C). Western blot analysis revealed that the expression levels of luteinization markers STAR, 3β-HSD, and HSD17B2, along with the cilia markers Arl13B and IFT88, were all significantly reduced (Figure 6D).

#### 2.3.2. The Expression of Luteinizing Related Molecules and the Secretion of Progesterone in Mouse Follicular Granulosa Cells Were Increased After the Induction of Primary Cilia

**Figure 7:**
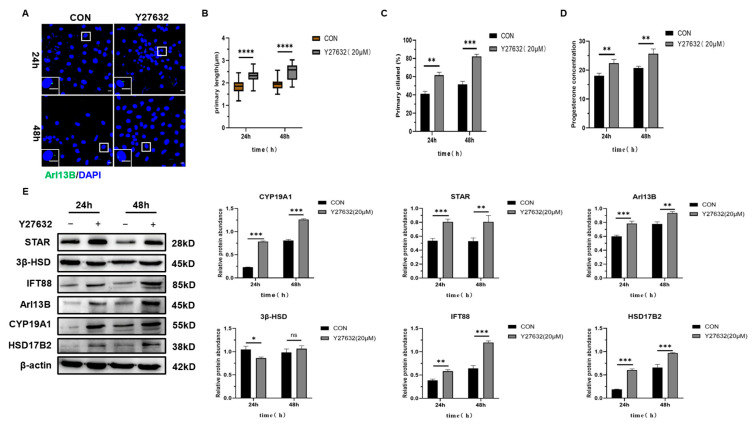
The effects of induced primary cilia formation on the luteinization of mouse granulosa cells. (**A**) Immunofluorescence staining of granulosa cells treated with 20 μM of Y27632 HCl for 24 and 48 h. Cell nuclei were stained with DAPI. Scale bar: 10 μm (insets and main panel). (**B**) The primary cilia length in granulosa cells treated with 20 μM of Y27632 HCl for 24 and 48 h. (**C**) The percentage of the primary cilia number in granulosa cells treated with 20 μM of Y27632 HCl for 24 and 48 h. (**D**) Progesterone secretion levels in granulosa cells treated with 20 μM of Y27632 HCl for 24 and 48 h. (**E**) Western blot analysis of STAR, 3β-HSD, IFT88, Arl13B, and CYP19A1 expression in granulosa cells treated with 20 μM of Y27632 HCl for 24 and 48 h. All data are expressed as the mean ± SD from three independent experiments, and representative images are shown. Statistical comparisons were performed using a two-tailed *t*-test. * *p* < 0.05, ** *p* < 0.01, *** *p* < 0.001, **** *p* < 0.0001, ^ns^ *p* > 0.05.

The ciliogenesis inducer Y27632 2HCl, a well-established agent known for its ability to stimulate primary cilia formation, was employed to investigate the potential role of primary cilia in the luteinization of granulosa cells. Granulosa cells were exposed to 20 μM of Y27632 2HCl for durations of 24 and 48 h. Immunofluorescence (Figure 7A) and ELISA analyses revealed that relative to the control group, treatment with 20 μM of Y27632 2HCl significantly increased the length and number of primary cilia (Figure 7B,C) and augmented progesterone secretion (Figure 7D). Furthermore, the expression levels of key luteinization-related molecules, including STAR, HSD17B2, CYP19A1, and the primary cilia proteins Arl13B and IFT88, were significantly elevated, whereas the expression of 3β-HSD remained unaffected (Figure 7E).

In conclusion, the data from Western blot, immunofluorescence, and ELISA analyses presented in Section 2.3.1 and Section 2.3.2 demonstrate that both the inhibition and induction of primary cilia formation exerted a significant impact on the luteinization process in mouse granulosa cells. The inhibition of ciliogenesis resulted in a delay in luteinization, whereas the induction of ciliogenesis expedited this process. These findings suggest that luteinization in mouse ovarian granulosa cells is positively correlated with the formation of primary cilia and that alterations in cilia length and number directly influence the synthesis and secretion of progesterone.

## 3. Discussion

Primary cilia, functioning as critical organelles in intercellular signal transduction, are intricately involved in a variety of signaling pathways that govern cellular communication. The signaling pathways mediated by primary cilia play pivotal roles in essential biological processes, including embryonic development, cellular polarization, and cell proliferation [37,38,39]. Recent advancements in the study of primary cilia have revealed their widespread presence across numerous mammalian species. Moreover, defects or the absence of primary cilia are implicated in a range of ciliopathies [40], including ovarian cancer, breast cancer, and prostate cancer. Prior investigations have demonstrated the presence of primary cilia on mouse ovarian granulosa cells, as observed under confocal microscopy [31]. However, the regulatory role and functional significance of primary cilia during granulosa cell luteinization remain unexplored. Both IFT88 and Arl13B are essential for ciliogenesis, with Arl13B being a ciliary membrane-specific protein that has been extensively utilized as a marker for cilia in various studies. Utilizing Arl13B, a primary cilia marker, we experimentally confirmed the presence of Arl13B in the ciliary membranes of mouse ovarian granulosa cells. Notably, during luteinization, primary cilia elongate and proliferate, suggesting that ciliogenesis may play a critical role in initiating granulosa cell luteinization. Arl13B serves not only as a marker for primary cilia but also plays a crucial role in the ciliogenesis process. In our study, we observed an upregulation in the expression of IFT88 and Arl13B proteins during the in vitro luteinization model, accompanied by increased progesterone secretion. This suggests that the elevated levels of these proteins may mediate the synthesis and secretion of progesterone.

The results of this study, which employed LH and INS injections to induce luteal formation in mouse ovaries, revealed that the upregulation of Arl13B expression in the corpus luteum is consistent with the changes observed in luteinization markers. As a critical structure for signal transduction, primary cilia’s changes in length and number may directly influence the activity of signaling pathways during luteinization. The localization and upregulation of Arl13B in the ovarian corpus luteum may reflect its regulatory role in luteal function during specific time periods. Moreover, its low expression in follicles may suggest that cilia play a limited role during follicular development. Both INS and LH significantly increase the expression of primary cilia proteins and promote P4 secretion, suggesting that cilia-mediated signaling pathways may be involved in LH- and INS-induced luteal formation stages.

Insulin, a protein hormone secreted by pancreatic β-cells, consists of insulin, the insulin-like growth factor (IGF), and their respective receptors. It plays a key role as the body’s principal hypoglycemic hormone. Recent studies have indicated that IGF-1, in conjunction with the follicle-stimulating hormone (FSH) and luteinizing hormone (LH), plays a pivotal role in regulating follicular development. It promotes granulosa cell proliferation and enhances the secretion of steroid hormones [41]. Nikola Sekulovski et al. ablated the insulin receptor (INS-R) and IGF receptor (IGF-R) in mouse ovaries, resulting in the severe disruption of both ovulation and luteal formation [42]. Research has demonstrated that IGF-1 accelerates the onset of STAR expression during in vitro luteinization, while also enhancing gonadotropin-induced STAR expression [43]. In mammals, ovulation is initiated following the LH surge, with the formation of the LH peak playing a crucial role in promoting both ovulation and luteal formation. Yuri Ko and Ju Hee Kim et al. have shown that insulin may enhance the role of LH in ERK1/2 phosphorylation, thereby exerting a positive effect on LH regulation [44]. Insulin exerts its effects by binding to insulin-like growth factor receptors, thereby promoting luteinization. Furthermore, primary cilia are closely associated with steroid hormones, suggesting an inherent link between primary cilia and luteinization. The exact role of primary cilia in this process, however, remains to be fully investigated.

IFT88, a key component of the intraflagellar transport complex in primary cilia, plays a critical role in ciliogenesis and the maintenance of ciliary function. A deficiency of IFT88 leads to ciliopathies [45]. Johnson et al. have shown that the synthesis and secretion of estrogen are impaired due to the absence of IFT88 in granulosa cells [31]. The corpus luteum, serving as the principal site for steroid hormone synthesis, is the primary source of both progesterone and estrogen production and secretion. Thus, we hypothesize that there is an inherent connection between primary cilia and luteinization. This study examined the dynamic changes in primary cilia during insulin-induced granulosa cell luteinization. The findings indicate that luteinization promotes ciliogenesis, and progesterone secretion gradually increases until it reaches a stable state, suggesting that primary cilia positively regulate progesterone secretion. Simultaneously, the expression levels of luteinization markers, including STAR and 3β-HSD, were elevated, while CYP19A1 expression was reduced. Moreover, the expression of primary cilia markers, such as IFT88 and Arl13B, exhibited a gradual increase. During ovulation after the surge of the LH, dynamic changes of steroid production occur in ovarian granulocyte cells. The rapid up-regulation of STAR and the rapid down-regulation of CYP19A1 in granulocyte cells undergoing luteinization during ovulation induced by the surge of the LH, and the changes in gene expression of STAR and CYP19A1 after the surge of the LH, effectively promote the production of progesterone [20]. Related studies have shown that epigenetic changes (histone modification and chromatin remodeling) occur in promoter regions of STAR and CYP19A1 in granulosae cells undergoing luteinization during ovulation [46]. Previous studies have reported that the elevation of STAR expression and the reduction in CYP19A1 expression serve as markers of luteinization [17,47]. The results of this study align with previous findings, supporting the notion that granulosa cells undergo luteinization and that primary cilia play a role in this process. However, limited research has demonstrated whether a direct connection exists between primary cilia and luteinization. This study demonstrates that primary cilia are present in mouse ovaries and actively participate in the luteinization process, playing a critical role in regulating ovarian development.

Given the dynamic changes in primary cilia during luteinization, we hypothesize that the synthesis and secretion of progesterone are intricately linked to the regulation of primary cilia. Previous studies have suggested that estrogen exerts an inhibitory effect on primary ciliogenesis in vivo [48]. However, primary ciliogenesis is concomitant with the secretion of progesterone [32]. Prior studies have demonstrated that both estrogen and progesterone play crucial roles in the luteinization process. However, the precise mechanisms through which they mediate their effects are yet to be elucidated. Consequently, the potential interaction between progesterone and estrogen in regulating primary ciliogenesis, as well as the signaling pathways through which they exert their effects, warrants further investigation. The results of this study indicate that progesterone secretion is regulated by primary cilia. Increasing the concentration of the ciliogenesis inhibitor CBA leads to a decrease in progesterone secretion, accompanied by the reduced expression of luteinization markers, including STAR, 3β-HSD, HSD17B2, CYP19A1, as well as primary cilia markers Arl13B and IFT88. Conversely, treatment with 20 μM of Y27632 HCl (a ciliogenesis inducer) led to increased progesterone secretion, along with the enhanced expression of luteinization markers and primary cilia proteins, including STAR, 3β-HSD, HSD17B2, CYP19A1, Arl13B, and IFT88. In other words, primary cilia regulate progesterone secretion. Building on previous studies, we propose that an interaction exists between estrogen, progesterone, and primary cilia, although the underlying mechanisms remain to be further elucidated. Consequently, future studies could investigate the interplay between steroid hormones and primary cilia to uncover the molecular mechanisms driving luteinization. Primary cilia may thus represent a novel avenue for exploring the pathophysiological mechanisms of follicular development and luteogenesis.

In summary, our study provides preliminary evidence that primary cilia play a role in regulating progesterone synthesis during luteinization in mouse ovarian granulosa cells. However, the molecular mechanisms by which primary cilia regulate ovarian function remain unclear, particularly regarding their role in modulating signaling pathways during granulosa cell luteinization, which warrants further investigation. Therefore, future studies will explore the molecular mechanisms by which primary cilia regulate ovarian function, identifying new targets and strategies that may offer novel approaches for treating and preventing miscarriage due to insufficient or dysfunctional luteal formation.

## 4. Materials and Methods

### 4.1. Animals and Ovarian Collection

In this study, 3-week-old, prepubertal female ICR mice, with an average body weight of approximately 25 g, were used as experimental animals. The mice were purchased from Guizhou Huigu Biotechnology Co., Ltd., Guiyang, China, and were housed at the Animal Center of Guizhou University. All experimental procedures involving mice were approved by the Animal Use and Management Committee of Guizhou University. The mice were kept under controlled conditions with a temperature of 25 °C and humidity levels ranging from 60% to 70%. The mice had free access to water and food.

To stimulate ovarian follicular development, immature female mice (21 days old) were intraperitoneally injected with 10 IU of pregnant mare serum gonadotropin (PMSG, Sanming Co., Ningbo, China). Mice were sacrificed by cervical dislocation at 48 h after PMSG administration. The ovaries were then collected for further experiments.

For the in vivo induction of ovarian luteinization in mice, the animals were divided into three groups: the control group (CON), the luteinizing hormone group (LH), and the insulin group (INS). After grouping the mice, they were injected with 10 IU of PMSG per animal at 16:00 on the same day. After 48 h of breeding, the CON group was injected with 200 μL of saline, the LH group with 8 IU of LH per animal, and the INS group with 1 IU/kg of insulin. Treatments were administered at 6, 12, 24, 48, and 72 h, with 3 mice per group at each time point. Blood serum and ovarian tissues were collected from each group at the specified time points for ELISA and Western blot (WB) analysis. ELISA was used to measure serum progesterone (P4) levels, while WB analysis was employed to examine the expression of luteinization-related proteins and primary cilia-associated molecules.

### 4.2. Cell Isolation, Culture, and Treatment

Cell culture protocol: The ultra-clean workbench was sterilized with UV light for 30 min. Twenty-one-day-old prepubertal female mice were intraperitoneally injected with 10 IU of PMSG per mouse, followed by cervical dislocation 48 h later. Using sterile scissors and forceps, the abdominal cavity was opened under aseptic conditions, and the ovaries were carefully collected and placed in physiological saline. The ovaries were promptly transported to the cell culture room and washed three times with PBS containing antibiotics. The ovaries were placed in a culture dish, and granulosa cells were released using two 1 mL syringes. The cells were centrifuged at 1500 rpm for 5 min, washed twice, resuspended, and seeded into DMEM-F12 complete medium containing 10% fetal bovine serum (FBS) and 1% penicillin–streptomycin. The cells were cultured in a CO_2_ incubator at 37 °C with 5% CO_2_ and saturated humidity. After 24 h, the medium was changed, and then again every two days thereafter. Cell samples and culture medium were collected for subsequent WB and ELISA assays.

For the in vitro granulosa cell luteinization model, ovarian granulosa cells were cultured in a DMEM-F12 medium supplemented with 10% fetal bovine serum (Z7010FBS-500, Xi’an Zhongtuan Biotechnology Co., Ltd., Xi’an China) and treated with insulin (2 μg/mL) for 6, 12, 24, and 48 h, and 1, 3, and 5 days. Cell samples and culture media were collected at each time point. Under in vitro luteinization conditions (2 μg/mL INS), the CBA co-treatment protocol was as follows: granulosa cells were treated with CBA (50 or 100 μM) under in vitro luteinization conditions (2 μg/mL INS) for 24 and 48 h, followed by the collection of cells and their culture medium for subsequent assays. For Y-27632 2HCl co-treatment under in vitro luteinization conditions, the protocol was as follows: granulosa cells were treated with Y-27632 2HCl (20 μM) under in vitro luteinization conditions (2 μg/mL INS) for 24 and 48 h, followed by the collection of cells and their culture medium for subsequent assays. All animal experiments were approved by the Animal Care and Use Committee of Guizhou University.

The reagents used for cell treatment included recombinant human insulin (2 μg/mL; PB180432, Procell, Wuhan, China), Y-27632 2HCl (20 μM; S8049, Selleck Chemicals, Shanghai, China), and CBA (50 μM or 100 μM; S8249, Selleck Chemicals).

### 4.3. ELISA Experiment

Prior to use, the ELISA kit was removed from 4 °C storage conditions and equilibrated at room temperature for 30 min. It was crucial to centrifuge the samples (serum and cell culture medium) and standards prior to measurement to ensure optimal accuracy. The number of wells to be used was determined, and any unused wells were stored in a sealed, dry bag at 4 °C. A blank well was set up, which did not contain any reagents, to serve as a control. A total of 50 μL of the gradient-diluted standard and 50 μL of the diluted samples (comprising 40 μL of PBS and 10 μL of the serum or cell culture medium) was added to to each well, followed by incubation at 37 °C for 30 min. After discarding the liquid, 300 μL of wash buffer was added to each well, and a 30 s wash step was performed and repeated five times. Following the final wash, the plate was inverted onto a clean tissue and gently tapped to remove any residual liquid. Subsequently, 50 μL of HRP-conjugate was added to each well, mixed thoroughly, the plate was sealed with adhesive film, and this was incubated at 37 °C for 30 min. The liquid was discarded from the wells, and then 300 μL of the wash buffer was added to each well, and a 30 s wash step was performed and repeated five times. After the final wash, the plate was inverted onto a clean tissue and gently tapped to remove any residual liquid. A total of 50 μL of Substrate A and Substrate B was added to each well, mixed thoroughly, and incubated at 37 °C, protected from light, for 15 min. A total of 50 μL of stop solution was added to each well, mixed thoroughly, and the OD450 value was measured within 15 min using a microplate reader (ELX88, Meigu Molecular Instruments, Shanghai, China).

The ELISA kits used in this study were purchased from Meimian Technology Co., Ltd. (MM-45704M1, Yancheng, China).

### 4.4. Western Blotting

Protein samples from cells were centrifuged at 13,000 rpm for 10 min at 4 °C. The supernatant was carefully collected and stored, and the protein concentration was determined using the BCA method. Based on the measured protein concentration, the samples were diluted with lysis buffer to the same concentration and mixed with the loading buffer. The samples were then boiled in a water bath for 5 min, after which they were vortexed and left at room temperature to prepare for loading. Electrophoresis: Equal volumes of the protein pre-stained marker and samples were loaded into the wells. The electrophoresis was run at a constant voltage of 80 V for 30 min, after which the voltage was increased to 120 V. The electrophoresis continued for 1–2 h, depending on the size of the target proteins. Transfer: Under an electric current, proteins were transferred from the gel to a PVDF membrane for 1.5 h. Blocking: After transfer, the membrane was incubated with 5% non-fat milk (A600669, Sangon, Shanghai, China) for 2 h to block nonspecific binding sites. The membrane was then incubated overnight at 4 °C with the primary antibody. Subsequently, the membrane was incubated with a horseradish peroxidase (HRP)-conjugated secondary antibody (1:5000; 32460, Invitrogen, Waltham MA USA) for 1 h. Signal Detection: The membrane was exposed to an ECL substrate (WBKLS0100, Millipore, Billerica, MA, USA) for signal visualization. The signals were detected using a Tanon imaging system (5200, Tanon, Shanghai, China). Images were collected and used for subsequent data analysis.

The primary antibodies used in this study include rabbit anti-Arl13B (1:2000; 17711-1-AP, Protein Tech, Guangzhou, China), rabbit anti-IFT88 (1:2000; 13967-1-AP, Protein Tech), rabbit anti-CYP19A1 (1:1000; PA1-21398, Invitrogen, Guangzhou, China), rabbit anti-HSD17B2 (1:1000; 10978-1-AP, Protein Tech, Guangzhou, China), rabbit anti-STAR (1:1000; 8449T; Cell Signaling, Guangzhou, China), and rabbit anti-3β-HSD (1:1000; 15516-1-AP; Protein Tech, Guangzhou, China).

### 4.5. Immunohistochemistry

(1) Granulosa cells were treated with 2 μg/mL insulin (INS), and cell coverslips were collected at 6, 12, 24, and 48 h. (2) Granulosa cells were co-treated with 2 μg/mL of INS and CBA (50 or 100 μM) under in vitro luteinization conditions for 24 and 48 h, with coverslips collected at these time points. (3) Granulosa cells were co-treated with 2 μg/mL of INS and Y-27632 2HCl (20 μM) under in vitro luteinization conditions for 24 and 48 h, with coverslips collected at these time points. Immunofluorescence (IF) was used to observe the morphological and quantitative changes of the primary cilia marker Arl13B at various time points. Fluorescence images were captured using a laser scanning confocal microscope at 60× magnification, ensuring identical parameter settings for all images.

Experimental procedure: The slides or coverslips were baked at 55 °C for 1 h, followed by deparaffinization (as per immunohistochemistry protocols) and antigen retrieval. The slides or coverslips were then blocked with 5% BSA at 37 °C for 1 h and incubated overnight with the primary antibody in a humidified chamber at 4 °C. The slides were then incubated with the appropriate secondary antibody at 37 °C for 30 min, followed by counterstaining with 4′,6-diamidino-2-phenylindole (DAPI) (D9542, Merck, Darmstadt, Germany) or propidium iodide (PI). Images were acquired using a Leica TCS SP 8 laser scanning confocal microscope (Leica Microsystems, Hesse, Germany). The ciliary membrane marker Arl13B was used for cilia measurement. For cilia analysis, Image J software (Image J v1.53k) was used to count ciliated cells and measure the cilia length.

The primary antibody used in this study was the rabbit anti-Arl13B antibody (1:2000; 17711-1-AP, Protein Tech).

### 4.6. Statistical Analysis

All experiments were conducted with at least three replicates. The results are expressed as the mean ± standard deviation (SD), unless otherwise stated. A one-way analysis of variance (ANOVA) was used to determine significant differences between groups, followed by a two-tailed unpaired Student’s *t*-test. A *p*-value of <0.05 was considered statistically significant. Statistical analysis was performed using GraphPad Prism version 8.2.0. All graphs were created using GraphPad Prism version 8.2.0.

## 5. Conclusions

In conclusion, we identified the presence of primary cilia in ovarian tissue and specifically on large luteal cells, and demonstrated their involvement in regulating the luteinization process of granulosa cells. Furthermore, our study demonstrated that during insulin-induced granulosa cell luteinization, primary cilia influence the expression of steroidogenic enzymes, thereby promoting the synthesis and secretion of progesterone.

## Data Availability

The original contributions presented in the study are included in the article, further inquiries can be directed to the corresponding author.

## Supplementary Materials

The following supporting information can be downloaded at https://www.mdpi.com/article/10.3390/ijms26052138/s1.

## References

[1] Imaimatsu K., Uchida A., Hiramatsu R., Kanai Y. (2022). Gonadal Sex Differentiation and Ovarian Organogenesis along the Cortical-Medullary Axis in Mammals. Int. J. Mol. Sci..

[2] Abedel-Majed M.A., Romereim S.M., Davis J.S., Cupp A.S. (2019). Perturbations in Lineage Specification of Granulosa and Theca Cells May Alter Corpus Luteum Formation and Function. Front. Endocrinol..

[3] Zachut M. (2015). Short communication: Concentrations of the mammalian lignan enterolactone in preovulatory follicles and the correlation with intrafollicular estradiol in dairy cows fed extruded flaxseed. J. Dairy Sci..

[4] Lalitkumar P.G.L., Lundström E., Byström B., Ujvari D., Murkes D., Tani E., Söderqvist G. (2023). Effects of Estradiol/Micronized Progesterone vs. Conjugated Equine Estrogens/Medroxyprogesterone Acetate on Breast Cancer Gene Expression in Healthy Postmenopausal Women. Int. J. Mol. Sci..

[5] Zhang X., Mi M., Hao W., Fan Q., Gao B. (2017). Progesterone down-regulates SLIT/ROBO expression in mouse corpus luteum. Acta Histochem..

[6] DeWitt N.A., Whirledge S., Kallen A.N. (2020). Updates on molecular and environmental determinants of luteal progesterone production. Mol. Cell Endocrinol..

[7] Przygrodzka E., Hou X., Zhang P., Plewes M.R., Franco R., Davis J.S. (2021). PKA and AMPK Signaling Pathways Differentially Regulate Luteal Steroidogenesis. Endocrinology.

[8] Brady K., Liu H.-C., Hicks J., Long J.A., Porter T.E. (2023). Global gene expression analysis of the turkey hen hypothalamo-pituitary-gonadal axis during the preovulatory hormonal surge. Poult. Sci..

[9] Stevenson H., Bartram S., Charalambides M.M., Murthy S., Petitt T., Pradeep A., Vineall O., Abaraonye I., Lancaster A., Koysombat K. (2022). Kisspeptin-neuron control of LH pulsatility and ovulation. Front. Endocrinol..

[10] Blitek A., Szymanska M., Pieczywek M., Morawska-Pucinska E. (2016). Luteal P4 synthesis in early pregnant gilts after induction of estrus with PMSG/hCG. Anim. Reprod. Sci..

[11] Hasegawa H., Nishimura R., Yamashita M., Yamaguchi T., Hishinuma M., Okuda K. (2019). Effect of hypoxia on progesterone production by cultured bovine early and mid luteal cells. J. Reprod. Dev..

[12] Lin Y.C., Cheung G., Porter E., Papadopoulos V. (2022). The neurosteroid pregnenolone is synthesized by a mitochondrial P450 enzyme other than CYP11A1 in human glial cells. J. Biol. Chem..

[13] Slominski R., Raman C., Elmets C., Jetten A., Slominski A., Tuckey R. (2021). The significance of CYP11A1 expression in skin physiology and pathology. Mol. Cell Endocrinol..

[14] Rajapaksha M., Kaur J., Prasad M., Pawlak K.J., Marshall B., Perry E.W., Whittal R.M., Bose H.S. (2016). An Outer Mitochondrial Translocase, Tom22, Is Crucial for Inner Mitochondrial Steroidogenic Regulation in Adrenal and Gonadal Tissues. Mol. Cell Biol..

[15] Orly J., Stocco D.M. (1999). The role of the steroidogenic acute regulatory (StAR) protein in female reproductive tissues. Horm. Metab. Res..

[16] Ma C., Rice I., Pen D., Li H., Cao G., Du C. (2016). The changes and signaling pathways of luteinization-related genes in the cyclic variation of sheep ovarian function. Chin. J. Vet. Sci. Anim. Husb..

[17] Johnson A.L., Lee J. (2016). Granulosa cell responsiveness to follicle stimulating hormone during early growth of hen ovarian follicles. Poult. Sci..

[18] Ronen-Fuhrmann T., Timberg R., King S.R., Hales K.H., Hales D.B., Stocco D.M., Orly J. (1998). Spatio-temporal expression patterns of steroidogenic acute regulatory protein (StAR) during follicular development in the rat ovary. Endocrinology.

[19] Hickey G.J., Chen S., Besman M.J., Shively J.E., Hall P.F., Gaddy-Kurten D., Richards J.S. (1988). Hormonal regulation, tissue distribution, and content of aromatase cytochrome P450 messenger ribonucleic acid and enzyme in rat ovarian follicles and corpora lutea: Relationship to estradiol biosynthesis. Endocrinology.

[20] Lee L., Asada H., Kizuka F., Tamura I., Maekawa R., Taketani T., Sato S., Yamagata Y., Tamura H., Sugino N. (2013). Changes in histone modification and DNA methylation of the StAR and Cyp19a1 promoter regions in granulosa cells undergoing luteinization during ovulation in rats. Endocrinology.

[21] Meng B., Cao Z., Gai Y., Liu M., Gao M., Chen M., Chen M., Ning Z., Luan X. (2019). Effects of recombinant goose adiponectin on steroid hormone secretion in Huoyan geese ovarian granulosa cells. Anim. Reprod. Sci..

[22] Long X., Chen L., Xiao X., Min X., Wu Y., Yang Z., Wen X. (2024). Structure, function, and research progress of primary cilia in reproductive physiology and reproductive diseases. Front. Cell Dev. Biol..

[23] Wang B., Liang Z., Liu P. (2021). Functional aspects of primary cilium in signaling, assembly and microenvironment in cancer. J. Cell Physiol..

[24] Delforge J.P., Thomas K., Roux F., de Siqueira J.C., Ferin J. (1972). Time relationships between granulosa cells growth and luteinization, and plasma luteinizing hormone discharge in human. 1. A morphometric analysis. Fertil. Steril..

[25] Yan Z., Neulen J., Raczek S., Weich H.A., Keck C., Grunwald K., Breckwoldt M. (1998). Vascular endothelial growth factor (VEGF)/vascular permeability factor (VPF) production by luteinized human granulosa cells in vitro; a paracrine signal in corpus luteum formation. Gynecol. Endocrinol..

[26] Zhan D., Xiang W., Guo F., Ma Y. (2017). Basic fibroblast growth factor increases IFT88 expression in chondrocytes. Mol. Med. Rep..

[27] Lu H., Toh M.T., Narasimhan V., Thamilselvam S.K., Choksi S.P., Roy S. (2015). A function for the Joubert syndrome protein Arl13b in ciliary membrane extension and ciliary length regulation. Dev. Biol..

[28] Shim S., Goyal R., Panoutsopoulos A.A., Balashova O.A., Lee D., Borodinsky L.N. (2023). Calcium dynamics at the neural cell primary cilium regulate Hedgehog signaling-dependent neurogenesis in the embryonic neural tube. Proc. Natl. Acad. Sci. USA.

[29] Miceli C., Roccio F., Penalva-Mousset L., Burtin M., Leroy C., Nemazanyy I., Kuperwasser N., Pontoglio M., Friedlander G., Morel E. (2020). The primary cilium and lipophagy translate mechanical forces to direct metabolic adaptation of kidney epithelial cells. Nat. Cell Biol..

[30] Meier-Vismara E., Walker N., Vogel A. (1979). Single cilia in the articular cartilage of the cat. Exp. Cell Biol..

[31] Johnson E.T., Nicola T., Roarty K., Yoder B.K., Haycraft C.J., Serra R. (2008). Role for primary cilia in the regulation of mouse ovarian function. Dev. Dyn..

[32] Li B., Yan Y.-P., Liang C., He Y.-Y., Wang Y., Li M.-Y., Chen S.-T., Li Y., Liu A.-X., Yan G.-J. (2022). Primary Cilia Restrain PI3K-AKT Signaling to Orchestrate Human Decidualization. Int. J. Mol. Sci..

[33] Geoghegan I.P., McNamara L.M., Hoey D.A. (2021). Estrogen withdrawal alters cytoskeletal and primary ciliary dynamics resulting in increased Hedgehog and osteoclastogenic paracrine signalling in osteocytes. Sci. Rep..

[34] Stocco C., Telleria C., Gibori G. (2007). The molecular control of corpus luteum formation, function, and regression. Endocr. Rev..

[35] Lv J. (2022). Effect of Insulin on Luteinization of Porcine Granule Cells. Ph.D. Thesis.

[36] Lv J., Cheng J., Zhang R., Zou C., An Q., Li P., Wei Y., Wei Y. (2022). Comparative study on methods of inducing luteinization of porcine granule cells in vitro. Chin. J. Anim. Sci..

[37] Guerra J., Chiodelli P., Tobia C., Gerri C., Presta M. (2020). Long-Pentraxin 3 Affects Primary Cilium in Zebrafish Embryo and Cancer Cells via the FGF System. Cancers.

[38] Atkins M., Wurmser M., Darmon M., Roche F., Nicol X., Métin C. (2023). CXCL12 targets the primary cilium cAMP/cGMP ratio to regulate cell polarity during migration. Nat. Commun..

[39] Toriyama M., Rizaldy D., Nakamura M., Atsumi Y., Toriyama M., Fujita F., Okada F., Morita A., Itoh H., Ishii K.J. (2023). Corrigendum: Dendritic cell proliferation by primary cilium in atopic dermatitis. Front. Mol. Biosci..

[40] Alzarka B., Charnaya O., Gunay-Aygun M. (2024). Diseases of the primary cilia: A clinical characteristics review. Pediatr. Nephrol..

[41] Wang Q., Yang X. (2017). Research progress of insulin-like growth factor I regulating follicle development. Chin. J. Eugen. Genet..

[42] Sekulovski N., Whorton A.E., Shi M., Hayashi K., MacLean J.A. (2020). Periovulatory insulin signaling is essential for ovulation, granulosa cell differentiation, and female fertility. FASEB J..

[43] Pescador N., Stocco D.M., Murphy B.D. (1999). Growth factor modulation of steroidogenic acute regulatory protein and luteinization in the pig ovary. Biol. Reprod..

[44] Ko Y., Kim J.H., Lee S.R., Kim S.H., Chae H.D. (2021). Influence of pretreatment of insulin on the phosphorylation of extracellular receptor kinase by gonadotropin-releasing hormone and gonadotropins in cultured human granulosa cells. Eur. J. Obstet. Gynecol. Reprod. Biol..

[45] Fitzsimons L.A., Tasouri E., Willaredt M.A., Stetson D., Gojak C., Kirsch J., Gardner H.A.R., Gorgas K., Tucker K.L. (2024). Primary cilia are critical for tracheoesophageal septation. Dev. Dyn..

[46] Sugino N. (2014). Molecular mechanisms of luteinization. Obstet. Gynecol. Sci..

[47] Strauss J.F., Christenson L.K., Devoto L., Martinez F. (2000). Providing progesterone for pregnancy: Control of cholesterol flux to the side-chain cleavage system. J. Reprod. Fertil. Suppl..

[48] Yan Y. (2020). Dynamic Changes and Regulation of Primary Cilia During Implantation and Decidualization of Mouse Embryos. Ph.D. Thesis.

