# Supplementary material for: The Role of Primary Cilia in Modulating the Luteinization Process of Ovarian Granulosa Cells in Mice
Source: Int J Mol Sci. 2025 Feb 27;26(5):2138. doi: 10.3390/ijms26052138 (PMC11900466; doi:10.3390/ijms26052138)
Supplement: Supplementary file 1 [file ijms-26-02138-s001.zip › ijms-3450870-supplementary.pdf]

Attachment S:

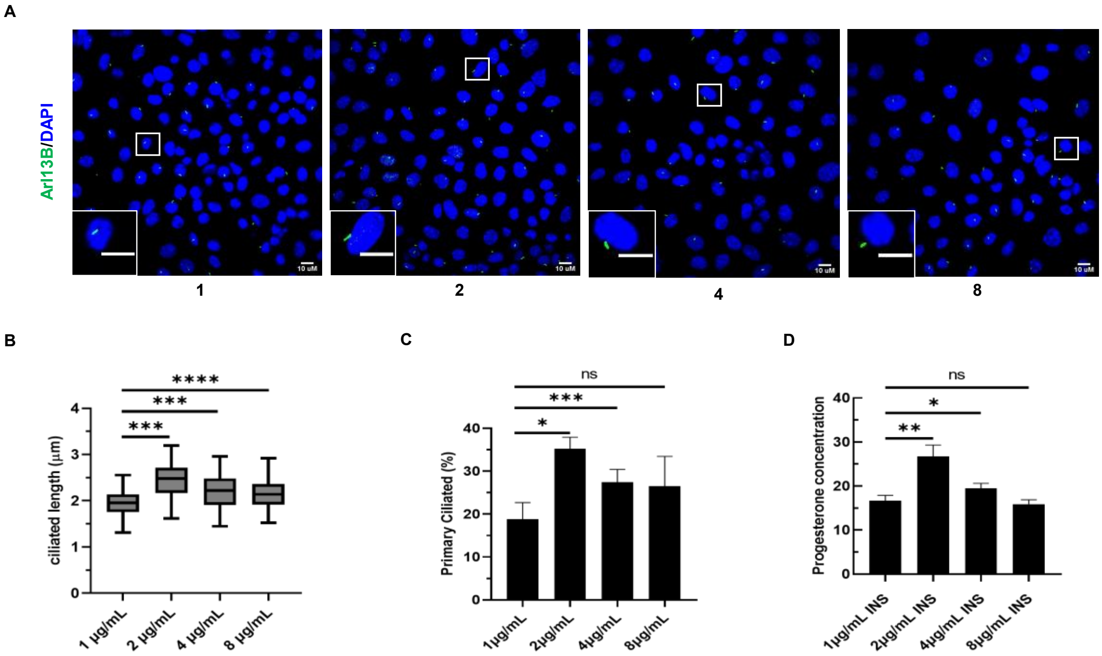

Figure S1. Effects of different concentrations of insulin on the number and length of primary cilia and progesterone secretion

(A) Immunofluorescence staining of granule cells after 24h treatment with different concentrations of insulin (1, 2, 4, 8μg/mL). Nuclei are labeled with DAPI. Scale bar: 10 μm (insets and main panel). (B) The effect of different concentrations of insulin (1, 2, 4, 8μg/mL) on the length of primary cilia of granulosa cells after 24h treatment. (C) Percentage of primary cilia of granulosa cells treated with different concentrations of insulin (1, 2, 4, 8μg/mL) for 24h. (D) Progesterone secretion in granulosa cells treated with different concentrations of insulin (1, 2, 4, 8μg/mL) for 24h. *P<0.05, ***P<0.001, ****P<0.0001, ^{ns}P>0.05.
